# Preoperative computed tomography-guided soft hook-wire localization for multiple pulmonary nodules

**DOI:** 10.3389/fonc.2025.1501165

**Published:** 2025-02-21

**Authors:** Guang-Chao Li, Zheng-Long Wu, Yi-Bing Shi, Jing-Ya Wang, Yu-Fei Fu

**Affiliations:** ^1^ Department of Radiology, Shanghai Sixth People’s Hospital, Shanghai, China; ^2^ Department of Radiology, Xuzhou Central Hospital, Xuzhou, China

**Keywords:** hook-wire, soft, localization, pulmonary nodule, multiple

## Abstract

**Background:**

While there have been prior reports on pulmonary nodule (PN) localization through computed tomography (CT)-guided soft hook-wire (SHW) placement, there remains a dearth of studies specifically focused on the CT-guided SHW localization of multiple PNs. This study was thus designed to specifically evaluate the efficacy and safety of this localization strategy in patients with multiple PNs.

**Methods:**

In total, 43 consecutive patients with multiple PNs underwent CT-guided SHW localization followed by video-assisted thoracoscopic surgery (VATS) to resent these PNs in our hospital between January 2022 and December 2022. A control group consisting of 140 individuals who underwent the CT-guided SHW localization of a single PN during the same interval was also selected. Both groups were analyzed retrospectively for safety and efficacy outcomes.

**Results:**

The final study population included 43 patients (94 PNs) in the multiple PN group, and 140 patients (140 PNs) in the single PN group, with all patients having undergone CT-guided SHW localization of these PNs. A one-stage procedure was employed for the localization of multiple PNs. The respective rates of technical success for SHW localization in the multiple and single PN groups were 98.9% and 100% (P = 0.402). The duration of localization in the multiple PN group was significantly longer than that in the single PN group (P = 0.001). The respective pneumothorax rates in the multiple and single PN groups were 32.5% (14/43) and 15.7% (22/140) (P = 0.015), while the corresponding lung hemorrhage incidence rates were 48.8% (21/43) and 36.4% (51/140) (P = 0.145). All PNs successfully underwent limited VATS resection, with a one-stage VATS procedure having been used for the resection of multiple PNs in a given patient.

**Conclusions:**

CT-guided SHW localization is a safe and effective means of localizing multiple PNs in a single patient. This simultaneous localization of multiple PNs can provide effective guidance for the subsequent one-stage limited VATS resection of these nodules.

## Introduction

Computed tomography (CT)-based screening is often used to evaluate patients facing a high risk of lung cancer, and it has been estimated to decrease lung cancer-associated mortality rates by 20% (1–3). Early-stage lung cancer is generally characterized by pulmonary nodules (PNs) detectable through CT scanning (1–3). While CT-based follow-up is generally employed for low-risk PNs (4), additional procedures are generally necessary for high-risk PNs, including CT-guided biopsy and video-assisted thoracoscopic surgery (VATS) resection (4). While CT-guided biopsy is a mini-invasive procedure with high levels of diagnostic accuracy, the rate of diagnostic failure for this procedure is still in the range of 10% (5).

Limited wedge or segmental VATS resection procedures remain the standard diagnostic approach for PNs, and in cases where PN staging is lower than invasive lung cancer, such resection can also be curative (6–8). To ensure that limited VATS resection is a success, clinicians have performed the CT-guided hook-wire (HW) localization of target PNs before VATS procedures (9–11). In patients with more than one high-risk PN, these multiple nodules can still be resected through a one-stage VATS procedure. In these cases, preoperative localization of the multiple nodules is particularly important, given that successful limited resection allows for the maximal preservation of respiratory function (12).

Conventional HW localization strategies generally lead to high rates of complications for patients harboring multiple PNs (13), with these rates reportedly rising as high as 64% (14). The primary cause of these complications is the rigidity of the HW material conventionally used in this approach. This issue has prompted some researchers to substitute soft HW (SHW) materials in place of the conventional rigid HW apparatus (15). While both such devices contain the hook structure, in the SHW version a soft suture is substituted in place of the stiff wire. This affords marked reductions in the incidence of localization-related complications (16). However, there has been little research to date specifically examining outcomes associated with the CT-guided SHW localization for multiple PNs.

This study was devised to evaluate the safety and clinical efficacy of the preoperative CT-guided SHW localization of multiple PNs.

## Methods

### Patients selection

This study was retrospective in design and received approval from the Ethics Committee of Xuzhou Central Hospital, and the Ethics Committee waived the requirement for written informed consent.

In total, 43 consecutive multiple PNs patients who received CT-guided SHW localization of these nodules followed by VATS resection between January 2022 and December 2022 were included in the present study. Inclusion criteria for these patients were as follows: (i) patients with more than one high-risk PN; (ii) patients with multiple unilateral PNs; (iii) the solid PNs with the > 10 mm PN-pleura distance, the GGNs regardless of PN-pleura distance; and (iv) patients 18-75 years of age. Patients were excluded if they exhibited (i) hilar PNs; (ii) PNs < 6 mm in diameter; and (iii) patients with severe cardiac, liver, kidney, pulmonary, or coagulatory disorders. As a control group, 140 patients who had undergone the CT-guided SHW localization for a single PN over the same period were also included in this study. High-risk PNs were assessed as per the Fleischner Society Guideline Recommendations (4).

### CT-guided SHW localization

**Figure 1:**
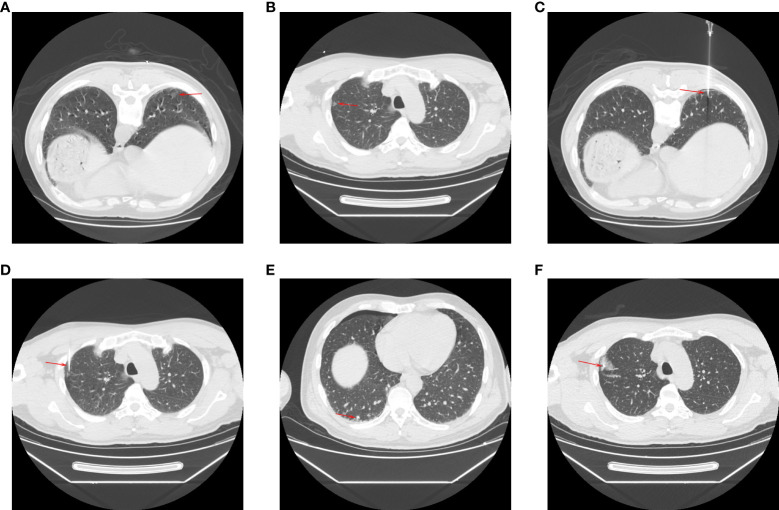
The procedures of CT-guided SHW localization for multiple PNs. The CT showed the PNs in **(A)** right lower (arrow) and **(B)** right upper (arrow) lobes. The needles **(C, D)** were inserted (arrows) near the PNs. The SHWs (e and f) were placed (arrows) near the PNs. **(E, F)** The intraoperative photos of the localization.

SHW localization procedures were performed with local anesthesia using a 16-row CT instrument (**Figures 1A, B**). The positioning of patients was selected according to target LN location and the related rib and vascular structures. The selection of the needle pathway was based on the position of the patient, selecting the pathway that minimized the distance between the skin and the target nodule. This procedure utilized a 20G guiding needle that was 10-15 cm in length (Senscure, Ningbo, China). This needle was placed into the lung lobe according to the selected needle pathway, and repeated CT scanning was used to confirm the position of needle tip. If the tip of the needle was located within 10 mm of the target nodule, SHW placement was performed (**Figures 1C, D**). During SHW placement, the anchor was inserted close to the target PN, and the needle was then removed, leaving the tri-colored suture attached to the anchor within the needle track while the distal suture end remained outside of the pleura. A one-stage procedure was used to localize multiple PNs in patients (**Figures 1E, F**). A final series of CT scans was used to evaluate patients for post-localization complications.

### VATS resection

All VATS procedures were performed via a standard two-incision approach under general anesthesia within 3 h of localization. For resection, patients were placed in the lateral decubitus position, after which a 3-5 mm incision through the 4/5th intercostal space was made along the anterior axillary line. The SHW localization marker was then used to guide limited resection. PN depth was evaluated based on preoperative CT images and the palpation of the SHW tip intraoperatively. A cutting suture device was used to perform limited resection based on PN depth. Segmental resection was performed if the margin was more than 2 cm from the edge of the PN, while wedge resection was performed in the remaining cases. Rapid pathological examination was performed for all resected PNs. In cases where PNs were diagnosed as invasive cancers, further lobectomy and lymphadenectomy were performed. For cases in which patients were diagnosed with mini-invasive cancer, lymph node sampling was performed. PNs staged at or below cancer *in situ* did not require any further procedures. A one-stage VATS procedure was used to resect all PNs. When patients had invasive tumors, lobectomy was performed for the nodule with the most advanced staging.

### Definitions

SHW localization procedures were defined as a technical success if (i) the SHW localization marker was visible while performing the VATS procedure, (ii) SHW dislodgement did not occur, and (iii) the target PN was located within the resected lung parenchymal tissue (wedge or segment). The duration of localization was calculated from the first to the last CT scans points. VATS time was measured as the interval between the first incision and wound closure.

Localization technical success was the primary endpoint for this study. Secondary endpoints included duration of localization, localization-related complications, VATS duration, surgery type, and final diagnosis.

### Statistical analyses

Normally distributed [skewed] data were reported as means ± standard deviations [medians], and were compared with independent sample t-tests [Mann-Whitney U tests]. Categorical data were reported as numbers (%) and compared with χ^2^ tests. Risk factors related to post-localization complications were detected through logistic regression analyses, with factors exhibiting significance in univariate analyses (P < 0.1) being incorporated into multivariate analyses. P < 0.05 was the threshold for significance, and SPSS 16.0 (SPSS Inc., IL, USA) was used to perform all analyses.

## Results

### Patients

A total of 43 patients underwent the CT-guided SHW localization of multiple PNs (94 total PNs) from January 2022 through December 2022, while 140 patients underwent SHW localization for single PNs over this same interval. One, 6, and 36 patients in the multiple PN group underwent the localization of 4, 3, and four 2 PNs. Baseline data for these patients are presented in **Table 1**.

**Table 1:** Comparison of patients’ baseline data between 2 groups.

	Multiple group	Single group	P value
Patients number	43	140	–
Age (years)	55.5 ± 8.8	55.7 ± 12.3	0.915
Gender			0.902
Male	14	47	
Female	29	93	
Previous tumor history	6	29	0.324
Smoking history	3	24	0.100
Pulmonary nodules number	94	140	Not applicable
Patients with 2 nodules	36	Not applicable	
Patients with 3 nodules	6	Not applicable	
Patients with 4 nodules	1	Not applicable	
Natures of the nodules			0.034
Solid	40	40	
Mixed GGN	6	20	
Pure GGN	48	80	
Nodules diameter (mm)	7.8 ± 2.4	8.4 ± 2.2	0.028
Nodule-pleura distant (mm)	10.7 ± 7.8	12.9 ± 10.2	0.068
Lobes			0.046
Left upper	17	28	
Left lower	10	31	
Right upper	36	52	
Right middle	6	7	
Right lower	28	22	

GGN, ground glass nodule.

### CT-guided localization

SHW localization achieved respective technical success rates of 98.9% and 100% in the multiple PN and single PN groups (P = 0.402). SHW dislodgement resulted in technical failure for one PN in a patient in the multiple PN group. The localization of multiple PNs was associated with the significant prolongation of localization time relative to that for a single PN (P = 0.001). In the multiple PN group, 23 patients underwent positional changes during localization (**Table 2**).

**Table 2:** Localization related results.

	Multiple group	Single group	P value
Localization successful rates	98.9% (93/94)	100% (140/140)	0.402
Localization time (min)	23.7 ± 11.7	9.0 ± 3.5	0.001
Position change (n)	23	Not applicable	Not applicable
Complications			
Pneumothorax	32.5% (14/43)	15.7% (22/140)	0.015
Lung hemorrhage	48.8% (21/43)	36.4% (51/140)	0.145

Pneumothorax rates in the multiple PN and single PN groups were 32.5% (14/43) and 15.7% (22/140), respectively (P = 0.015). Longer localization time was identified as being independently associated with the risk of pneumothorax through univariate and multivariate analyses (**Table 3**).

**Table 3:** Predictors of pneumothorax for multiple pulmonary nodules localization.

Variables	Univariate analysis			Multivariate analysis		
	Hazard ratio	95% CI	P value	Hazard ratio	95% CI	P value
Age	0.945	0.874-1.022	0.158			
Gender						
Male	1					
Female	1.316	0.328-5.280	0.699			
Smoking history						
No	1					
Yes	0.000	0.000	0.999			
Tumor history						
No	1					
Yes	2.364	0.411-11.584	0.335			
Lung side						
Right	1					
Left	0.889	0.219-3.609	0.869			
Same lobe						
Yes	1					
No	1.271	0.340-4.754	0.722			
Number of nodules	2.010	0.504-8.016	0.323			
Minimum nodule diameter	1.499	0.878-2.557	0.138			
Maximum nodule-pleura distant	1.058	0.973-1.151	0.185			
Localization time	1.062	1.001-1.127	0.045	1.062	1.001-1.127	0.045
Position change						
No	1					
Yes	1.929	0.518-7.174	0.327			

Lung hemorrhage rates in the multiple PN and single PN groups were 48.8% (21/43) and 36.4% (51/140), respectively (P = 0.145). No independent risk factors for lung hemorrhage were successfully identified (**Table 4**).

**Table 4:** Predictors of lung hemorrhage for multiple pulmonary nodules localization.

Variables	Univariate analysis			Multivariate analysis		
	Hazard ratio	95% CI	P value	Hazard ratio	95% CI	P value
Age	1.014	0.946-1.087	0.693			
Gender						
Male	1					
Female	1.429	0.395-5.163	0.586			
Smoking history						
No	1					
Yes	0.500	0.042-5.966	0.584			
Tumor history						
No	1					
Yes	1.056	0.188-5.926	0.951			
Lung side						
Right	1					
Left	0.857	0.233-3.159	0.817			
Same lobe						
Yes	1					
No	1.667	0.484-5.736	0.418			
Number of nodules	1.697	0.415-6.938	0.461			
Minimum nodule diameter	1.050	0.644-1.713	0.844			
Maximum nodule-pleura distant	1.052	0.970-1.140	0.223			
Localization time	1.023	0.970-1.079	0.404			
Position change						
No	1					
Yes	0.629	0.188-2.103	0.452			

No localization-associated complications resulted in subsequent VATS resection procedures being delayed or otherwise affected. Therefore, the localization-associated complications were not specially managed.

### VATS resection

Limited VATS resection procedures were successful for all target PNs among patients in this study (**Table 5**). Even though one nodule experienced technical failure for the SHW localization procedure in the multiple PN group, limited resection was successfully performed as the site of the needle puncture remained visible in the visceral pleura intraoperatively while performing VATS resection. In all instances, a one-stage VATS procedure was used to resect multiple PNs. Both groups were comparable in terms of limited VATS resection types (P = 0.577) and VATS duration (P = 0.200). Three of the patients in the multiple PN group exhibited multiple invasive adenocarcinoma lesions, and in these cases, additional lobectomy was performed for the most advanced lesion. **Table 5** presents the pathological diagnoses for all lesions in this study.

**Table 5:** VATS related results.

	Multiple group	Single group	P value
VATS time (min)	104.4 ± 63.0	91.6 ± 55.3	0.200
Blood loss (ml)	25 (Q1: 17.5; Q3: 50)	20 (Q1: 10; Q3: 50)	0.213
Types of limited resection			0.577
Wedge	89	130	
Segmental	5	10	
Additional lobectomy	9	45	0.001
Pathological diagnoses			0.007
Invasive adenocarcinoma	12	45	
Mini-invasive adenocarcinoma	30	42	
Adenocarcinoma in site	17	20	
Precancerous lesion	9	5	
Benign	26	28	

VATS, video-assisted thoracoscopic surgery.

## Discussion

Of patients exhibiting high-risk PNs, 22.2%-24.5% present with more than one high-risk nodule (17, 18). In these patients with multiple PNs, advantages to CT-guided one-stage localization procedures include (i) the ability of one-stage procedures to enable VATS resection through a one-stage procedure, obviating the requirement for stages resection, and (ii) one-stage localization procedures can aid in the resection of all PNs with a minimal resection range.

In this study, both the safety and efficacy of the CT-guided SHW localization of ipsilateral multiple PNs were assessed. This approach yielded respective technical success rates for the localization procedure itself and subsequent limited VATS resection of 98.9% and 100%. Relative to the localization of a single PN, localizing multiple PNs required more time but was still an effective one-stage localization procedure in these patients, facilitating the one-stage limited VATS resection of multiple target nodules.

Pneumothorax and lung hemorrhage rates following the localization of multiple PNs have previously been reported to range from 27.5%–56.8% and 18.9%–60%, respectively (9, 17, 19). The corresponding rates in this study (32.5% and 48.8%) fell within this range. The high complication rates were mainly attributed to that the multiple PNs localization required more needle puncture procedures. While these complications were more common among patients who underwent the localization of multiple PNs, none resulted in any delays or other changes in the VATS resection procedures, emphasizing the safety of CT-guided SHW localization for multiple PNs.

Coil and liquid-based materials have also been employed to localize multiple PNs (17–19). Liquid-based materials are inexpensive, have a good safety profile, and can be readily deployed, but they suffer from diffusion such that localization failure can arise in some cases. Coil localization is a more complex procedure as a consequence of the shape of the coil and the requirement that its distal end be proximal to the PN while its proximal end extends beyond the visceral pleura (19). Coils also suffer from the risk of unintentionally being inserted fully into the parenchyma of the lung. In such cases, fluoroscopy-based VATS procedures must be conducted such that both patients and the surgeons themselves are exposed to radiation.

VATS procedure durations and blood loss volumes were similar in both patient groups (P = 0.200 and 0.213). This may be related to the fact that the rate of invasive adenocarcinoma was lower in the multiple PN group relative to the single PN group (12.8% vs. 32.1%, P = 0.007), and invasive adenocarcinomas generally necessitate additional lobectomy. In addition, this study included 3 patients with multiple invasive adenocarcinoma lesions, and additional lobectomy was only performed for the most advanced lesion in these cases. As a result, the rate of additional lobectomy was reduced in the multiple PN group relative to the single PN group (9.6% vs. 32.1%, P = 0.001).

There are certain limitations to this study, the most prominent of which is the retrospective study design. As the number of PNs per patient cannot be controlled, performing a randomized controlled trial was not possible. Secondly, some of the baseline data were not balanced between patient groups, including PN etiology, location, and diameter, potentially contributing to additional bias. Third, no comparisons were made between SHW and other localization materials in this patient population, underscoring the need for future comparative analyses aimed at establishing the optimal materials for the localization of multiple PNs.

## Conclusion

In summary, CT-guided SHW localization is a safe and effective means of localizing multiple PNs in a single patient. This simultaneous localization of multiple PNs can provide effective guidance for the subsequent one-stage limited VATS resection of these nodules.

## Data Availability

The raw data supporting the conclusions of this article will be made available by the authors, without undue reservation.
